# [Expression of Concern] The inhibitory roles of Ihh downregulation on chondrocyte growth and differentiation 

**DOI:** 10.3892/etm.2025.12946

**Published:** 2025-08-13

**Authors:** Ang Deng, Hongqi Zhang, Minyu Hu, Shaohua Liu, Yuxiang Wang, Qile Gao, Chaofeng Guo

Exp Ther Med 15:789-794, 2018; DOI: 10.3892/etm.2017.5458

Following the publication of this paper, it was drawn to the Editor’s attention by a concerned reader that, for the Von Kossa staining experiments shown in Fig. 3B, the ‘Blank’ and the ‘NC’ panels appeared to be overlapping, suggesting that data which were intended to show the results from differently performed experiments had apparently been derived from the same original source. The authors were contacted by the Editorial Office to offer an explanation for the apparent anomaly in the presentation of the data in this paper, although up to this time, no response from them has been forthcoming. Owing to the fact that the Editorial Office has been made aware of potential issues surrounding the scientific integrity of this paper, we are issuing an Expression of Concern to notify readers of this potential problem while the Editorial Office continues to investigate this matter further.

